# The Paradox of NET Involvement in the Pathogenesis of Inflammatory Bowel Disease

**DOI:** 10.1093/ibd/izaf283

**Published:** 2025-11-18

**Authors:** Harriet Comer-Calder, Hassan O J Morad

**Affiliations:** Neuroscience, Inflammatory Disorders & Therapeutics Research Group, Centre for Nutraceuticals, School of Life Sciences, University of Westminster, London, United Kingdom; Neuroscience, Inflammatory Disorders & Therapeutics Research Group, Centre for Nutraceuticals, School of Life Sciences, University of Westminster, London, United Kingdom

**Keywords:** neutrophil, inflammatory bowel disease, IBD, NETs, inflammation

## Abstract

Inflammatory bowel disease (IBD), namely Crohn’s disease (CD) and ulcerative colitis (UC), are defined by chronic, non-resolving inflammation of the intestinal mucosa. Neutrophils are the first responders in inflammation, executing various effector functions, including chemotaxis, phagocytosis, degranulation and the release of cytokines, reactive oxygen species (ROS) and neutrophil extracellular traps (NETs). Amongst all neutrophil functions, emerging evidence increasingly suggests that NET release may be particularly relevant in underpinning the pathogenesis of IBD. NETs are extracellular structures composed of chromatin, antimicrobial proteins, and oxidative enzymes released by neutrophils to trap and neutralize pathogens. In this review, we discuss the protective roles of NETs in intestinal health and how, under tight physiological regulation, they can prevent pathogenic invasion, exert anti-inflammatory effects, and play an important role in wound healing and intestinal tissue repair. Conversely, we consider how inflammation-driven changes in neutrophil activation, phenotype and immunometabolism can cause dysregulation in NET production and clearance and lead to harmful intestinal effects that can prolong intestinal and chronic inflammation in IBD. Specifically, we explore how uncontrolled NET production can damage intestinal epithelial integrity, increase bacterial translocation and increase thromboembolic risk, ultimately linking NETs to the pro-inflammatory pathogenesis of IBD.

Key Messages **What is already known?**

Neutrophils are important immune cells in the regulation of gut health and homeostasis.

**What is new here?**

Under tight physiological regulation, neutrophil extracellular traps (NETs) can prevent pathogen dissemination in the gut, exert anti-inflammatory effects, and promote intestinal tissue repair, however, within the pro-inflammatory environment of inflammatory bowel disease (IBD), uncontrolled NET production and delayed removal can prolong the chronic inflammation central to Crohn’s disease and ulcerative colitis.

**How can this study help patient care?**

An understanding of the inflammation-driven changes in neutrophil activation, phenotype and immunometabolism in IBD that lead to excessive NET release can help the design and development of therapeutics which reduce NET release in IBD to healthy, inflammation-resolving levels seen in the healthy gut.

## Introduction

Neutrophils are the most abundant immune cells in human blood, making up 50-70% of circulating leukocytes. They are the first line of defense against invading pathogens and are the primary mediators of the innate immune system.^1^ Neutrophils have four main killing mechanisms, including phagocytosis, degranulation, release of reactive oxygen species (ROS) and, importantly, the formation of neutrophil extracellular traps (NETs). Beyond this, neutrophils undergo chemotaxis and produce cytokines that modulate inflammation, interact with other immune cells and regulate wound healing.^2^

Also called polymorphonuclear (PMN) leukocytes, neutrophils differentiate and develop in the bone marrow by a process known as granulopoiesis, before entering the bloodstream in their quiescent state where they typically survive for less than 24 h.^3^ Under homeostatic and local inflammatory conditions, neutrophils exit the blood by extravasation and migrate to sites of inflammation and/or infection in the tissues, where they become activated for an acute, short-lived response before removal by other neutrophils or macrophages. However, when neutrophil activation is uncontrolled and prolonged, immune mediated tissue damage can occur. This can cause and/or be a symptom of chronic inflammation that can be associated with autoimmune and inflammatory diseases.^4^

One such inflammatory disease associated with neutrophil-mediated chronic inflammation is inflammatory bowel disease (IBD). This review will discuss the roles of neutrophils in inflammation during IBD, with particular focus on the important pathophysiology of NETs within the disease.

## Inflammatory Bowel Disease

IBD is a generic term encompassing various gastrointestinal inflammatory conditions, namely Crohn’s disease (CD) and ulcerative colitis (UC). IBD is a chronic inflammatory disorder characterized by repetitive episodes of inflammation thought to be caused by an abnormal immune response to intestinal microbiota.^5^

There are 6.8 million people living with IBD worldwide, shown epidemiologically by an ever-increasing prevalence, static incidence, high mortality, morbidity and low quality of life.^6^^,^^7^ Both CD and UC are lifelong, relapsing conditions with cyclical periods of flare and remission.^8^ No curative treatment is available, with current treatment modalities primarily including immunomodulators, biologics, and monoclonal antibodies.^9^

**Figure 1.**
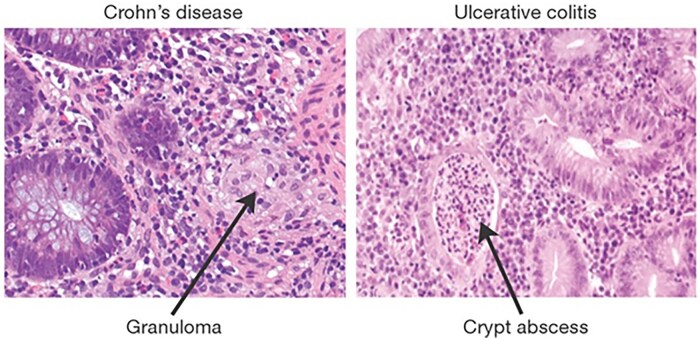
Immunomorphology of Crohn’s disease (CD) and ulcerative colitis (UC). Hematoxylin and eosin stained colonic cells showing histological hallmarks of active CD (left) and UC (right). CD: histological biopsy analysis on the left shows a well-defined non-caseating granuloma containing macrophages, giant cells, and epithelioid cells. A halo of lymphoid cells surrounds the granuloma, showing neutrophil infiltration (but not necrosis) in the mucosa. UC: many neutrophils are visible on the right within the lamina propria and crypts, where they collect to form necrotic abscesses typical of active UC. Original figure by Xavier and Podolsky,^18^ reproduced with permission under license.

CD is characterized by deep transmural inflammation that can occur anywhere from mouth to anus, presenting discontinuously in patches of inflammation known as “skip lesions.”^10^ CD most commonly localizes in the terminal ileum, leading to nutrient malabsorption and anemia.^11^ Macroscopically, CD is associated with ulcers, transepithelial fissures, and protective extraintestinal fat wrapping, and serious complications of stricture and fistula formation can occur.^12^ Thickening of the muscularis mucosae occurs due to a repeated cycle of fissuring and repair that causes a “cobble-stoning” appearance.^13^ On histological examination, mucosal crypt architecture distortion, deep transepithelial ulcers, hallmark non-caseating granulomas, and extensive mucosal neutrophil infiltration are visible, highlighting the strong immunological basis of CD pathogenesis (Figure 1). ^14^

UC is the most common form of IBD worldwide. Associated with ulceration and friable necrotic loss of epithelium that occurs exclusively in the large intestine, UC results in superficial erosions, colonic bleeding, crypt abscesses, and pseudo-polyp formation (Figure 1).^15^ Unlike in CD, inflammation (driven largely by neutrophils) is restricted to the mucosa and submucosa of the colon and occurs continuously.^16^ Chronic mucosal inflammation leads to progressive scarring, loss of haustral markings, and hypertrophy of the muscularis mucosae, leading to radiographic findings of a “lead pipe colon” due to its smooth, rigid appearance.^17^

In both CD and UC, a loss of tolerance to commensal microflora is thought to be a key trigger for initiating homeostatic imbalance and excessive mucosal immunity, establishing an autoimmune-type response and perpetuating pathogenic inflammation.^18^ Whilst each disease has distinct intestinal epithelial manifestations (Figure 1), both involve an inappropriate immune response, chronic unresolved inflammation and intestinal damage.^19^ The exact etiology of both conditions is contested, but a complex interplay between genetic predisposition, intestinal dysbiosis, mucosal barrier dysfunction, and an aberrant immunological response is suspected.^14^

Neutrophils are central to the chronic-relapsing inflammation that underscores the immunological basis of both CD and UC. Of all the effector mechanisms of neutrophils, there is increasing evidence that NET release is central to the pathogenesis of IBD, responsible for perpetuating chronic inflammation and large deterioration of gut health.^20^ However, it is important to note that neutrophil effector functions, including NETs, have positive, protective roles in gut health under normal physiological conditions.^21^ The paradoxical contributions of neutrophils and NETs to gut protection and gut deterioration in IBD will be reviewed in the following sections.

### The Protective Roles of Neutrophils in the Gut during Homeostasis and Local Inflammation

While neutrophils were originally described as homogenous effector cells associated with infection, the discovery of neutrophils at sterile injuries in the absence of infection challenged this view, and more recent studies have shown neutrophils to display incredible heterogeneity in which environmental stimuli can alter their phenotype and function.^22^ In the gut, it is now known that neutrophils play a complex homeostatic role in the delicate mediation between intestinal epithelial cells (IECs), microbes and intestinal immunity. Neutrophils act protectively in the gut to maintain this equilibrium and defend the mucosa from invading luminal pathogens.^23^ They are also involved in maintaining the balance between response and toleration toward intestinal contents and commensal microbiota.^24^

### Protective Effector Functions of Neutrophils: Chemotaxis, Cytokines/Chemokines, Phagocytosis, ROS, and Degranulation

**Figure 2.**
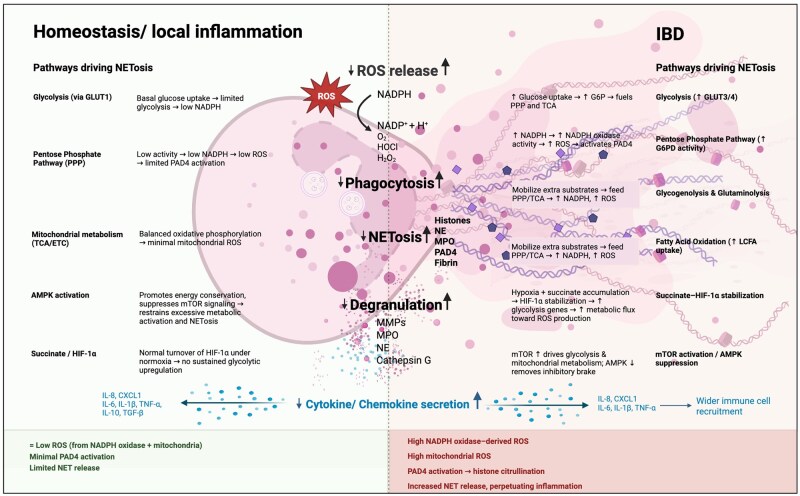
Neutrophil effector functions in homeostasis/local inflammation and IBD. Summary of neutrophil effector functions including cytokine secretion (leading to chemotaxis), phagocytosis, degranulation, ROS release and NET formation in homeostasis/local inflammation and IBD. Neutrophils secrete cytokines and chemokines which can activate/inactivate other neutrophils and other immune cells, affecting their phenotype and directly modulating their chemotaxis. In IBD pro-inflammatory cytokine/chemokine secretion increases, while anti-inflammatory cytokine/chemokine secretion decreases. During the process of phagocytosis, NADPH oxidase generates ROS within the phagolysosome. These ROS can also be released extracellularly. NADPH oxidase activity increases in IBD leading to increased ROS release. Degranulation occurs by releasing primary, secondary, and tertiary granules in the phagosome and extracellularly, with degranulation increasing in IBD. NETosis; the release of DNA and histones stimulated by NADPH oxidase-derived ROS activating PAD4, occurs at low levels in homeostasis/local inflammation, but dramatically increases in IBD. The immunometabolism pathways driving the levels of NETosis under both states are detailed. Up and down arrows indicate an increase or decrease in function or pathway. Abbreviations used in figure: IBD (inflammatory bowel disease), ROS (reactive oxygen species), NADPH (nicotinamide adenine dinucleotide phosphate), H (hydrogen), $O_{2}^{-}$ (superoxide), HOCl (hypochlorous acid), H_2_O_2_ (hydrogen peroxide), NET (neutrophil extracellular trap), NE (neutrophil elastase), MPO (myeloperoxidase), PAD4 (protein arginine deiminase 4), MMP (matrix metalloproteinase), IL (interleukin), CXCL1 (C-X-C motif chemokine ligand 1), TNF (tumor necrosis factor), TGF (transforming growth factor), GLUT (glucose transporter protein), PPP (pentose phosphate pathway), TCA (tricarboxylic acid cycle), ETC (electron transport chain), AMPK (AMP-activated protein kinase), AMP (adenosine monophosphate), HIF (hypoxia-inducible factor), mTOR (mammalian target of rapamycin), G6P (glucose-6-phosphate), G6PD (flucose-6-phosphate dehydrogenase), LCFA (long-chain fatty acid). Original figure created in BioRender. Comer-Calder, H. (2025) https://BioRender.com/y3uptow.

Neutrophils arrive in the gut following extravasation from blood vessels and successful chemotaxis to the tissues. Here, neutrophils then phagocytose microorganisms through a process of actin cytoskeleton reorganization, trapping the pathogen within the phagosome (Figure 2). Assembly of nicotinamide adenine dinucleotide phosphate (NADPH) oxidase complex and generation of ROS (Figure 2) creates a powerful antimicrobial environment that has been shown to play an important role in homeostasis of intestinal microbiota in murine models.^25^ Neutrophil phagocytosis is also involved in removal of necrotic tissue and cellular debris, playing an important role in intestinal epithelial wound healing.^26^ Degranulation refers to the sequential release of membrane and intracellular granules of increasing antimicrobial effects from tertiary, secondary to finally primary granules containing the most powerful primary granules with neutrophil elastase (NE), myeloperoxidase (MPO), cathepsin G, defensins and permeability increasing proteins^27^ (Figure 2). Degranulation can occur extracellularly, or within the enclosed phagosome which concentrates the granule effects whilst limiting host tissue damage, ensuring intestinal epithelial integrity is protected.^28^

Activated neutrophils also produce cytokines in a tightly regulated manner*,* stimulating an acute immune response against an intestinal pathogen (Figure 2).^29^ Synthesis and release of pro-inflammatory cytokines, such as tumor necrosis factor (TNF)-α, interleukin (IL)-6 and IL-1β activate further neutrophils, priming them to induce a pro-inflammatory phenotype through up-regulation of receptors, such as Toll-like receptor (TLR)4, C5a receptor (C5aR)1 and C-X-C motif chemokine receptor (CXCR)1 and CXCR2.^30^ The release of chemokines, such as IL-8 and CXCL1 generates gradients for the pro-inflammatory primed neutrophils to sense and migrate toward (chemotaxis) in order to arrive at the site of pathogenic attack.^31^ Contrastingly, the release of anti-inflammatory cytokines, such as IL-10 and transforming growth factor (TGF)-β suppresses pro-inflammatory TNF-α and acts as a key immunoregulator in the intestines to limit the inflammatory response and collateral damage to host tissues.^32^ At the end of their lifespan, apoptotic neutrophils are immunosuppressive and are associated with the release of further anti-inflammatory IL-10 and growth factor TGF-β and reduced TNF-α.^33^

### Protective Effector Function of Neutrophils: NETs

NET formation is the release of extracellular deoxyribonucleic acid (DNA) fibers embellished with microbicidal proteins and oxidative enzymes that act as sticky traps to catch and immobilize the pathogen (Figure 2).^34^ There are a diverse range of stimuli for NET production, including pathogen associated molecular patterns (PAMPs) such as bacterial components like lipopolysaccharide (LPS), β-glucan or flagellin, and damage associated molecular patterns (DAMPs) such as high mobility group box 1 (HMGB1) protein and cold-inducible ribonucleic acid (RNA) binding protein (CIRP).^35^ In addition, cytokines such as TNF-α have a stimulating effect on NET formation, whereby inhibiting TNF-α results in fewer colonic NETs and related proteins.^36^ Mostly, NETosis is suicidal (lytic) to the neutrophil due to the lytic release, but amazingly vital (non-lytic) NETosis also occurs, in which nuclear DNA remains but mitochondrial DNA is expelled to scavenge the pathogen.^37^

Lytic NET formation occurs slowly, stimulated within three to 4 h by the invading pathogen itself, LPS, cytokines such as IL-8, or synthetically by phorbol 12-myristate 13-acetate (PMA). Increased intracellular calcium activates NADPH oxidase complexes via protein kinase C (PKC) or the rapidly accelerated fibrosarcoma (RAF)-mitogen-activated protein kinase (MEK)-extracellular signal-regulated kinase (ERK) pathway, generating ROS and activating peptidyl arginine deiminase-4 (PAD4).^38^ PAD4 is a key regulator of NETosis, causing histone citrullination and translocation of NE and MPO into the nucleus to cause chromatin decondensation ahead of the nuclear and subsequent outer membrane rupture to release the chromatin extracellularly.^39^ Recently, it has been discovered that NE converts Gasdermin D into its active form to facilitate pore formation within the nuclear and plasma membrane to enhance NET release.^40^ Non-lytic NETosis occurs more rapidly, within an hour after stimulation following TLR and complement activation. This leads to a rapid increase in cytosolic calcium, triggering PAD4 histone citrullination and chromatin decondensation independently from NADPH-oxidized ROS.^41^ However, unlike in lytic NET formation, the decondensed chromatin is extruded via vesicles, leaving the plasma membrane intact.^42^

Production of NETs by neutrophils prevents pathogenic invasion in the intestine, for example a clear correlation between NET generation and clearance of *C. rodentium* intestinal infections is documented in mouse models.^43^ Conversely, inhibition of NET formation has been shown to increase bacterial translocation, suggesting their importance in preventing bacterial dissemination in the gut.^44^ Given the hypotheses regarding microbial dysbiosis as a potential driver of IBD, protective roles of NETs in clearance of certain bacterial strains and prevention of bacterial translocation is highly relevant. NETs can also induce granule exocytosis and ROS production in a self-amplification mechanism that enables a coordinated neutrophil response against an intestinal pathogen.^45^

In addition to the beneficial antibacterial action of NETs, they can also reduce intestinal damage caused by inflammation. There is increasing evidence that the constituent proteases may have anti-inflammatory properties under certain physiological conditions, reducing the secretion of dendritic and macrophage cytokines.^46^ Furthermore, the formation of NET aggregates is linked to a reduction of cytokines and chemokines in mouse models of gout, suggesting inflammation-resolving properties.^47^

Neutropenic patients often suffer from reduced wound healing.^48^ Research has long focused on the negatives of NETs with wound healing, but more recently, attention has turned to their beneficial role, which may address this apparent clinical dichotomy. The ability of NET aggregations to act as inflammatory mediators, degrading cytokines and chemokines through proteolytic activity, supports their role in the resolution of inflammation and wound healing.^47^ Aggregated NETs can also sequester and detoxify extracellular histones released from necrotic cells and even from NETs themselves at the site of mucosal injury, limiting the chronification of their histone-induced cytotoxic effects and promoting healing.^49^ NET aggregates have also been shown to encapsulate necrotic wound tissue, creating a barrier between viable tissues and necrotic areas, preventing the spread of inflammation and preserving healthy residual tissue.^50^ Inflammatory tissue damage stimulates the production of a proangiogenic subset of neutrophils, cluster of differentiation (CD)11b+/granulocyte receptor (Gr)-1+CXCR4 high, which are recruited up the chemotactic gradient toward the site of mucosal injury by vascular endothelial growth factor A (VEGF-A) and chemokine (C-X-C motif) ligand (CXCL)12.^51^ Furthermore, matrix metalloproteinase (MMP)-9 has proangiogenic properties; collectively, these neutrophils and constituent enzymes stimulate angiogenesis, facilitating oxygen and nutrient supply and localized immune cell infiltration required for intestinal tissue repair.^52^ Mucosal healing is the clinical endpoint of many IBD treatments, highlighting the importance of furthering our understanding of the beneficial side of NETs in wound healing for future therapeutics.

### The Pathogenic Roles of Neutrophils in IBD

When neutrophils and their mechanisms are not tightly regulated, their beneficial role in maintaining intestinal homeostasis can tip over into a detrimental one, contributing toward chronic inflammation, such as is seen in IBD. Neutrophil over-activity and/or dysfunction can cause intestinal inflammation. Neutrophil involvement in CD and UC may differ in that consistent excessive activation is associated with UC, whereas fluctuating periodic excessive activation, leading to defective functionality is observed in CD.^53^^,^^54^ Both ultimately lead to a dysregulated inflammatory response.^55^ Neutrophil infiltration and IBD are so intimately connected that the primary biomarker for disease severity is calprotectin, a protein released by activated neutrophils.^56^

### Damaging Neutrophil Effector Functions in IBD: Chemotaxis, Cytokines/Chemokines, Phagocytosis, ROS, and Degranulation

While we have discussed the physiological functions of neutrophil chemotaxis and effector mechanisms, including phagocytosis, ROS, cytokine release, and degranulation in maintaining intestinal homeostasis, when these processes are not tightly regulated, they can lead to persistent neutrophil activation and intestinal damage that underpins the chronic, non-resolving inflammatory pathogenesis of IBD.

Large-scale neutrophil transepithelial migration in IBD disrupts intestinal epithelial integrity, leading to increased bacterial translocation and a positive-feedback loop of inflammation characterized by the activation of MMP, a disintegrin and metalloprotease (ADAM)17, TNF-α, and harmful ROS, which contribute to crypt distortion in active IBD.^14^ Increased neutrophil release of pro-inflammatory cytokines, such as IL-8, activate further neutrophil chemotaxis and lead to the overactivation of the immune response (Figure 2). High IL-8 levels are associated with active UC due to the increased hyper-inflammatory neutrophil recruitment, explaining its continuous inflammatory presentation, while fluctuating levels of IL-8 in CD can lead to dysfunctional, irregular chemotaxis and the cyclical inflammation that presents.^57–59^ Increased pro-inflammatory cytokine release (and reduced anti-inflammatory cytokine release) drives recruitment and activation of other immune cell types amplifies pro-inflammatory signaling networks in both the innate and adaptive immune system, perpetuating profound intestinal inflammation (Figure 2).^23^

Excess ROS formation, both extracellularly and as a by-product of disproportionate phagocytosis (Figure 2), contributes to oxidative stress and mucosal damage.^60^ Chronic oxidative stress induces DNA damage and lipid peroxidation, increasing epithelial permeability and facilitating pathogen invasion that constantly reintroduces inflammatory PAMPS.^61^ More specifically, the NADPH oxidase complex produces large amounts of superoxide ($O_{2}^{-}$), a precursor for hydrogen peroxide (H_2_O_2_), which directly damages epithelial tight junctions.^62^ Moreover, extracellular ROS release also has a secondary role in recruiting further neutrophils, through H_2_O_2_, to the site of infection by activating neutrophil transient receptor potential cation channel, subfamily M, member 2 (TRPM2), contributing to further intestinal inflammation.^63^ Degranulation of cytotoxic granules, such as MMP-9, degrades collagen in intestinal tissue and releases proline-glycine-proline (PGPs), leading to increased neutrophil recruitment and a cycle of chronic inflammation (Figure 2).^64^ MMPs can also activate the release of cytokines, such as IL-8, and degrade cadherins, inhibiting epithelial function and mucosal healing.^65^

### Damaging Neutrophil Effector Function in IBD: NETs

Whilst the proposed beneficial antibacterial and anti-inflammatory effects of NETs were discussed in the previous section, NETs are also thought to play an important role in the pathogenesis of IBD (Figure 2). Several recent studies show increased NET formation in both UC and CD models compared to experimental controls.^66–69^ Other studies found that NET abundance was more significantly increased in UC than in CD, perhaps due to the continuous nature of the inflammatory phenotype of UC and/or the cellular dysfunction in CD.^36^^,^^70^ Increased NET formation has now been demonstrated in colonic biopsy sections, fecal samples and blood samples of human patients with active IBD.^54^ PAD4 is an accurate biomarker of NETosis and is seen proportionately raised in samples with high NET formation in IBD.^71^ Due to increased PAD4 activity causing increased citrullination of arginine residues on histone H3, serum citrullinated histone 3 can also be used as a concurrent biomarker of NETosis along with PAD4.^72^^,^^73^ Furthermore, NET constituent proteins such as NE, MPO, cathepsin G, defensins and others have been shown by liquid chromatography-mass spectrometry (LCMS)–based IBD proteomics studies,^74^^,^^75^ immunofluorescence,^36^^,^^70^ immunohistochemistry,^36^ western blot,^36^^,^^70^ and capture enzyme-linked immunosorbent assay (ELISA).^66^^,^^76^ The wide range of visualization techniques and the diversity of human sample type indicates the strength of collective evidence for increased NETs in IBD. However, many NET-associated proteins can exist independently of NET extrusion and therefore studies using this as evidence for increased NETosis need to be evaluated carefully.^54^ A particular neutrophil phenotype, CD177+ is observed at higher levels in the intestinal mucosa of IBD patients.^66^ CD177+ neutrophils are “NET-prone,” producing a greater number of NETs than CD177- neutrophils, contributing to NET abundance observed in IBD. Importantly, the number of CD177+ neutrophils correlates to the disease activity index of both CD and UC, linking NET abundance and IBD disease severity.^66^

Unregulated NET production is involved in IBD pathogenesis in several ways. NETs promote intestinal epithelial breakdown, enhancing bacterial translocation and increasing inflammation.^76^ NET proteins such as NE disrupt tight junctions between epithelial cells, degrading key junctional proteins like E-cadherin, causing cell detachment and compromising epithelial barrier function.^77^ NET-associated histones disrupt the integrity of tight junctions and adherens junctions and induce intestinal epithelial cell death, resulting in heightened permeability of the gut epithelium and further damage to the mucosal barrier in IBD.^78^ Damage to the intestines caused by NETs leads to the release of DAMPs, such as HMGB1. These serve as pro-inflammatory signals that trigger additional NET formation, creating a positive feedback loop that sustains chronic inflammation.^79^ Dinallo et al. demonstrated that increased NETs are associated with increased cytokines, such as TNF-α, IL-6, IL-8 and IL-1β in human lamina propria cell cultures.^36^ The ability of NETs to upregulate pro-inflammatory cytokines ultimately leads to excessive neutrophil infiltration in the intestinal mucosa responsible for crypt abscesses and ulcer formation in CD and UC, respectively.^36^ Furthermore, IL-8 and IL-6 stimulate further neutrophil recruitment and trigger further NET release, perpetuating the NET-induced inflammatory damage in IBD (Figure 2).^80^

A lesser-known aspect of IBD is the significant increase in thromboembolic risk. An exciting area of research is the hypothesis that NETs provide a scaffold for erythrocyte and platelet adhesion, possibly explaining the prothrombotic tendency in IBD.^81^ This idea has recently been highlighted by the observation that deoxyribonuclease (DNase) I treatment reduced thrombotic features in a dextran sulphate sodium (DSS) IBD model.^76^ Patients with IBD are three times more likely to experience thromboembolic events compared to the general population, with strong evidence linking NETs to this thrombotic tendency in IBD.^82^ The structural framework of NETs facilitates the accumulation of platelets, erythrocytes, fibrinogen, platelet adhesion factors, and other prothrombotic elements.^83^ Additionally, histone components, specifically H3 and H4 of NETs, initiate platelet activation and enhance blood coagulation via TLR2 and TLR4 signaling pathways.^84^ NETs trigger increased exposure of endothelial phosphatidylserine expression, promoting the assembly of coagulation complexes and increasing thrombin production.^85^ NE on NETs breaks down and inactivates tissue factor (TF) pathway inhibitors, removing the brake on coagulation regulation while simultaneously activating the coagulation cascade’s extrinsic pathway via NET protease activity.^86^ Supporting studies show that when platelets from healthy patients are incubated with NETs derived from patients with active UC and CD, there is a 32% increase in procoagulant activity and a 42% rise in fibrin formation.^66^ Not only does this result in a risk of severe IBD extraintestinal complications, including venous and arterial thromboembolism, but hypercoagulation itself is a pro-inflammatory state, shown increasingly to contribute to the inflammatory pathogenesis of IBD.^87^

The balance of NET degradation and production is key to maintaining NET homeostasis. Impaired NET clearance undermines the resolution of intestinal inflammation, chronically inducing the release of pro-inflammatory cytokines and damaging the integrity of the intestinal mucosal barrier, perpetuating the inflammatory response.^88^ Physiologically, NETs are degraded by DNA degradation enzymes, with reduced enzymatic activity correlated with NET accumulation in autoimmune diseases.^89^ DNase and exonucleases three prime Repair exonuclease (TREX)1 and TREX2 are primarily responsible for breaking down the DNA and structural disruption of NETs. Macrophages employ DNase I to extracellularly digest NETs, degrading phagocytosed NETs in their lysosomal space.^90^ However, in IBD, DNase I activity has been found to be significantly reduced compared to healthy controls.^91^ Furthermore, impaired NET degradation is observed more specifically in patients with active UC, suggesting the constant hyper-inflammatory environment suppresses the anti-inflammatory mechanisms which restore homeostasis.^69^ Reduced DNase I activity and increased NET abundance found concurrently in active IBD points to the significance of impaired NET clearance in the pathogenesis of IBD.

### Recent Advancements and Areas for Future Research in NETs and IBD

#### Omics, Neutrophil Heterogeneity, Subsets, and Granulocytic Myeloid-Derived Suppressor Cells

Neutrophil heterogeneity is increasingly recognized as central to IBD pathogenesis, with advances in single-cell and spatial omics revealing transcriptionally distinct subsets anatomically primed to drive NET-mediated inflammation.^92^

At the systemic level, Neuenfeldt et al. identified a C-C chemokine receptor (CCR)5^+^ TNF-receptor 2-driven neutrophil subset in the blood and lamina propria of UC patients.^93^ Single-cell transcriptomics revealed a reduced antibacterial profile but enhanced NETotic potential, shown by the localization of NE within the nucleus, significant ROS-MEK–ERK signaling, and transcriptional bias toward PAD4-dependent NETosis.^94^ Mechanistically, CCR5^+^ neutrophils undergo a three-hit priming sequence: interferon (IFN)-γ-induced TNF expression, TNF-receptor-2-driven NE upregulation, and ROS amplification, collectively driving spontaneous NET formation.^93^ Resulting NET release and associated NE disrupt epithelial barriers, amplifying mucosal inflammation and positioning CCR5+ as a transcriptionally primed driver of NET-induced inflammation in UC.^88^ Interestingly, anti-TNF exposure increased NET formation in this subset, offering a mechanistic link to therapy resistance and paradoxical worsening of some patients following biologic treatment.^95^ These findings suggest CCR5^+^ pro-NETotic neutrophils may act as a significant upstream driver of systemic immune activation and mucosal injury in IBD, representing a potential therapeutic target via CCR5^+^ antagonism or PAD4 inhibition.^96^^,^^97^

Once recruited into the colon, CCR5^+^ precursors seed spatially defined neutrophil niches. Yalom et al. used spatial transcriptomics to identify lamina propria neutrophils (LPNs) and epithelium-associated neutrophils (EANs) found within UC biopsies.^98^ EANs, at the crypt surface, decreased during active colitis (in both UC patients and murine models), while LPNs deeper within the lamina propria persisted even during remission, suggesting functional divergence.^98^ Integrated spatial and single-cell transcriptomics, combined with intravital microscopy, confirmed these distinct roles by showing that LPNs exhibited chemotactic and phagocytic behaviors consistent with immune surveillance, whilst EANs showed heightened NET release, ROS and TNF-α secretion, acting as primary cytotoxic effectors that perpetuate mucosal injury.^98^ This microanatomical stratification highlights the significance of biopsy location, informs biomarker development and treatment monitoring, and suggests EANs as potential therapeutic targets for precision intervention in IBD.

Complementary analysis of ulcerated mucosa by Garrido-Trigo et al. identified three distinct neutrophil states in IBD using single-cell and CosMx spatial imaging.^99^ Multi-omic data revealed N1 and N3 subsets within ulcer beds and crypt abscesses displayed heightened NETosis and tissue injury. ­Conversely, N2, found within the ulcer stroma, displayed low levels of NETosis but contributed to stromal remodeling and angiogenesis, suggesting a role in fibrosis and chronic inflammation in IBD.^100^^,^^101^

Alongside classical neutrophil subsets, granulocytic myeloid-derived suppressor cells (gMDSCs) represent another NET-producing population in IBD. Patients with UC and CD show increased accumulation of gMDSCs in peripheral blood compared to healthy subjects, with frequency correlating with disease severity.^102^ While originally considered as immunosuppressive through inhibition of T-cells, macrophages, and natural killer (NK) cells, their role in IBD is more complex. Studies suggest that in the inflamed mucosa, gMDSCs undergo pathological reprogramming, losing suppressive capacity and instead driving T-cell expansion and amplifying chronic inflammation.^103^ Multi-omic profiling further reveals their divergence from neutrophils, with distinct kinase signatures, cell-cycle and autophagy programming, reduced nuclear factor kappa-light-chain-enhancer of activated B cells (NF-κB)/MEK signaling, and unique surface markers such as lectin-like oxidized low-density lipoprotein receptor (LOX)-1 alongside altered chemokine receptor expression.^104^ Functionally, these differences are likely to translate to altered NET protein composition, although any contribution toward mucosal pathology remains to be completely defined.

Under inflammatory conditions, gMDSCs can differentiate into dendritic-like cells, upregulate inducible nitric oxide synthases (iNOS), and release ROS/nitric oxide (NO), fueling T-helper (Th)17 polarization and epithelial disruption.^105^ In addition, gMDSCs-derived pro-inflammatory mediators, such as IL-6, TNF-α, granulocyte-macrophage colony-stimulating factor (GM-CSF), and CXCL1, amplify inflammation by recruiting further immune cells and reinforcing a strong inflammatory feedback loop.^106^ Collectively, these findings suggest that, in addition to contributing to NET-induced inflammation, gMDSCs drive the continual reinitiation of intestinal inflammation, serving as a possible mechanistic link between acute inflammation and the persistence of chronic disease in IBD.^107^

Together, these transcriptomic studies reveal a continuum of functionally distinct neutrophil states in IBD. The layered heterogeneity contributes to the complex pattern of acute flare and chronicity observed in IBD, emphasizing the central role of NETs as a converging effector of pathogenesis and target for precision modulation.^95^^,^^108^ Looking ahead, the integration of machine learning with transcriptomics has the potential to refine neutrophil state classification, linking NET signatures to therapeutic responses and enabling an increasingly personalized and targeted therapeutic approach to IBD.^109^

#### Trained Immunity

Beyond static heterogeneity, emerging evidence suggests that neutrophils can acquire trained immunity, characterized by epigenetic and metabolic rewiring that lowers NETosis thresholds and amplifies their response upon re-exposure to stimuli. Whilst first studied in monocytes, both central bone marrow progenitor and peripheral tissue imprinting mechanisms are now recognized in neutrophils.^110^^,^^111^ Functionally, this follows a “first hit/second hit” model, whereby priming stimuli such as GM-CSF, TNF-α, C5a lead to a poised state, biasing neutrophils toward hyper-responsiveness when re-stimulated.^112^^,^^113^ Converging epigenetic, metabolic and mitochondrial ROS signaling cascades reduce activation thresholds and bias neutrophils toward PAD4 and Gasdermin D-dependent NETosis.^114^^,^^115^

In IBD, trained immunity provides a plausible explanation for sustained NETosis within inflamed mucosa, with accumulation previously attributed mainly to reduced DNase I-mediated clearance.^95^ Instead, sustained inflammatory priming may lower NETosis thresholds, perpetuating inflammation, fueling the vicious cycle of relapse and therapeutic resistance.^36^^,^^116^ Supporting this, neutrophils in diabetes exhibit trained immunity, where metabolic reprogramming primes neutrophils for NETosis, impairing wound healing but reversible with targeted inhibition.^117^ Therapeutically, this positions anti-NET approaches such as PAD4 inhibition, Gasdermin D blockade, DNase-mediated clearance or C5aR1 antagonism as possible “de-training” methods, potentially resetting maladaptive neutrophil memory, blocking sustained priming rather than simply suppressing downstream NET-induced inflammation.^113^^,^^115^^,^^118^

#### Immunometabolism

Immunometabolism in neutrophils was previously thought to be driven solely by glycolysis, however, more recent discoveries have shown the involvement of several interlinked pathways.

In glycolysis, neutrophils take up glucose primarily through glucose transporter protein (GLUT) 1.^119^ Glucose is converted into glucose-6-phosphate (G6P), which, through a multi-step process, generates pyruvate that drives a net gain in adenosine triphosphate (ATP) for cellular function. Pyruvate can also be decarboxylated to form acetyl-coenzyme (co)A, which can enter the tricarboxylic acid (TCA) cycle and through oxidative phosphorylation in the electron transport chain (ETC) in the neutrophil mitochondria synthesize further ATP.^120^

However, in inflammatory microenvironments, an increase in neutrophil energy demand for processes such as chemotaxis and phagocytosis drives increased glucose uptake through GLUT3 and GLUT4.^121^ A concurrent upregulation of G6P dehydrogenase activates the pentose phosphate pathway (PPP) leading eventually to the generation of ribose-5-phosphate and NADPH.^122^ The subsequent activation of NADPH oxidases then release ROS. While these ROS have functions in neutrophil activation and the microbiocidal oxidative bust, they are also key activators of PAD4 and histone citrullination, which prime neutrophils for NETosis (Figure 2).^39^ Due to inflammation-induced glycolysis depleting glucose levels, neutrophils can deploy glycogenolysis to generate glucose from stored glycogen,^123^ but can also utilize glutamine immunometabolism.^124^ Up taken glutamine is enzymatically converted to α-ketoglutaric acid (AKG) and NADPH. While AKG can then propagate the TCA, the NADPH-mediated increase in NADPH oxidase activity is exploited by PPP for further ROS and subsequent NET production.^125^^,^^126^ Thus, modulation of immunometabolism pathways driven by energy demands in response to inflammation can prime neutrophils for increased NET release (Figure 2).

Studies show that activation of neutrophils by mediators present in IBD, such as LPS^127^ and TNF-α^128^ can significantly promote glutamine uptake, ultimately leading to NETosis through PPP glycolysis pathway exploitation.^129^ The centrality of the interlocking immunometabolism pathways described above is shown by Rodríguez-Espinosa et al., whereby inhibiting glycolysis and glutaminolysis through 2-deoxyglucose (2-DG) suppressed NET formation in human neutrophils *in vitro*,^126^ and in a model of mucosal inflammation inhibition with the same compound prevented NET release in mice *in vivo.*^130^

Where increased inflammation-driven glycolysis has depleted glucose, neutrophils can employ autophagy-mediated breakdown of internal lipids through fatty acid oxidation (FAO) to generate acetyl-coA for the TCA (Figure 2).^131^ In IBD, however, there is a relationship between increased long-chain fatty acids (LCFAs) in the gut and condition severity and increased neutrophil up take of external LCFAs and subsequent FAO in IBD contributes to overactivity of the ETC, stimulating NETosis (Figure 2).^132^^,^^133^ Interestingly, supplementation of short-chain fatty acids in place of LCFAs reduces intestinal inflammation in animal models.^134–136^

In IBD, increased oxygen use by immune cells, and inflammation-induced vasculitis can lead to a hypoxic state.^137^ This activates hypoxia-inducible factors (HIFs), such as HIF1-α (HIF1A), which can promote an upregulation in glycolytic genes, increasing glycolysis, and as a result NETosis.^138^ Conversely, knockdown of HIF1A decreases NET release in human neutrophils^139^ and inhibition of HIF1A with dihydromyricetin reduces NET release and inflammation in mouse IBD models.^140^ Induction of HIF1A expression^141^ and overall increased neutrophil metabolic activity is controlled through mammalian target of rapamycin (mTOR) activation.^142^ mTOR signaling allows an increase in glycolysis and mitochondrial metabolism^131^ and knockdown with rapamycin reduces NET activity.^139^ mTOR signaling is usually inhibited by adenosine monophosphate (AMP)-activated protein kinase (AMPK) phosphorylation,^143^ promoting energy-conservation, but in IBD, AMPK phosphorylation decreases and mTOR signaling is activated^144^ as has been shown in animal studies (Figure 2).^145^

The immunometabolite succinate can also accumulate in high concentrations in the IBD gut, due to microbiota shifts promoting overgrowth of bacteria that produce more succinate from dietary fiber.^146^ Succinate up take by neutrophils can inhibit degradation of HIF1A, leading to its stabilization (Figure 2).^147^ While this promotes NET release through increased glycolysis, it can also lead to increased IL-1β production, perpetuating inflammation.^148^ Succinate can also accumulate intracellularly in neutrophils in IBD due to hypoxia-induced ETC retardation, leading to a reversal in succinate dehydrogenase function and the mitochondrial ETC reversing from ATP generation to ROS production^149^ causing further NETosis through PAD4 activation (Figure 2).^39^ Increased NET release through succinate accumulation has been shown in mouse inflammatory models.^150^ Succinate stabilization of HIF1A has been shown continue even when inflammation has subsided and normoxic conditions have returned.^151^ Thus, this may be an underlying factor driving metabolic priming in IBD, where chronic inflammatory exposure and altered immunometabolism biases neutrophils toward a pro-NET state. However, significant gaps in the literature remain linking metabolic studies of neutrophils in IBD to distinct neutrophil subsets mentioned previously, or how metabolic rewiring integrates with trained immunity to reduce NETosis thresholds.

#### Therapeutics

The recognition that neutrophil subsets, including CCR5^+^ pro-NETotic cells, EANs, and N1-3 states, share NET formation as a convergent effector pathway has driven interest in therapies that directly modulate NETosis in IBD. Multiple drug classes are now in clinical development for inflammatory diseases, with translational potential for IBD.^54^

PAD4 inhibition disrupts histone citrullination and chromatin condensation, essential for NET formation.^152^ The pan-PAD inhibitor Cl-amidine effectively suppressed colitis in DSS murine models, both prophylactically and therapeutically, reducing NET burden, lowering NET marker citrullinated histone H3, restoring barrier integrity and reducing mucosal inflammation mirrored in PAD4 knock-out phenotypes.^153^^,^^154^ The selective PAD4 inhibitor GSK484 has also been shown to reduce mucosal NET density, although biomarker validation remains limited.^155^

Gasdermin D inhibition represents an alternative therapeutic approach, targeting the lytic extrusion of NETs.^156^ Mechanistically, Gasdermin D acts as a central integration point of pro-inflammatory cell death: caspase-dependent pyroptosis and NE-mediated cleavage that activates the pore-forming N-terminal fragment required for lytic NETosis.^40^ Pharmacologically, the FDA-approved, repurposed drug Disulfiram prevents Gasdermin D pore formation and prevents pyroptotic NETosis, with sepsis and corona virus disease of 2019 (COVID-19) clinical trials showing effective reduction in NET burden and improved clinical outcomes.^157^ In IBD, spatial transcriptomic profiling has demonstrated lytic NET release within barrier-disruptive EANs and N1-3 ulcer-associated subsets, highlighting the therapeutic potential of Gasdermin D blockade on mucosal injury and inflammation.^40^^,^^109^ In parallel, small molecule inhibitor LDC7559 selectively binds Gasdermin D, blocking both pyroptosis and NETosis in neutrophils, reducing inflammatory pathology without decreasing protective phagocytic functions. Supporting studies further show that Gasdermin D deficiency prolongs neutrophil lifespan, whilst the N-terminal fragment has direct antimicrobial activity, reinforcing Gasdermin D as a selective target that not only reduces NET-induced inflammation but has additional protective and anti-inflammatory properties.^158^^,^^159^

Complement is a double-edged regulator of inflammation in IBD, with C1q, CD46, CD55, CD59, and C6 emerging as protective against intestinal inflammation, while C3, C5, and C5a amplify mucosal injury via pro-inflammatory cascades.^160^ C5a, in particular, mediates neutrophil activation and is a potent inducer of NETosis, with complement blockade of the C5a-C5aR1 axis preventing the priming and recruitment of NET-prone neutrophils.^161^^,^^162^ Transcriptomic analysis confirms upregulation of C5aR1 in active UC lesions, reinforcing its mechanistic relevance to NET-induced inflammation in IBD pathology.^160^ A recent study on the inhibition of C5aR1 with nano-encapsulated PMX205 significantly reduced pre-clinical colitis with efficacy comparable to C5aR1-deficient models.^163^ These findings support the argument for targeted complement blockade as a therapeutic avenue for precision NET-mediated inflammation modulation in IBD.

Finally, another approach is to target NET clearance, rather than inhibiting their formation or release. Recombinant DNase I, which catabolizes the DNA scaffold of NETs, is already widely used to treat cystic fibrosis due to its ability to reduce sputum viscosity and NET-induced airway inflammation.^164^ In pre-clinical colitis models, nano-enzymatic DNase delivery significantly improved clinical outcomes, limiting weight loss, preserving colon length, reducing pro-inflammatory cytokine levels, and maintaining mucosal integrity. It also reduced colonic neutrophil recruitment and NETosis, directly linking clearance of NETs to disease attenuation.^165^ Despite its tolerability and FDA-approved status, the short plasma half-life of DNase-I and the potential for mucosal injury from exposed histones and proteases released during NET degradation remain a therapeutic limitation.^166^ Furthermore, prolonged NET clearance poses a risk toward host defense, highlighting the need for strategies to balance pathogenic NET removal with preservation of protective antibacterial function, particularly used adjuvantly with immunosuppressive IBD therapies.^167^

## Conclusion

Neutrophils have a central role in innate immunity and act as a double-edged sword in the intestines, restoring intestinal homeostasis and killing invading pathogens but also participating in the dysregulation of immune response and chronic inflammation associated with IBD. This chronic, perpetuating inflammation in IBD is driven to a large extent by NETs, which can cause damage and degradation of the gut when their release is not properly regulated. IDB is a life-long condition with no cure. Currently, management by anti-inflammatory/immunosuppressive therapies and biologics, which are primarily TNF-α inhibitors,^168^ is the major clinical intervention in IBD. Significant focus on neutrophil mechanisms, such as NETs in this review, but also chemotaxis, cytokine release, phagocytosis, degranulation and ROS production, alongside analysis of neutrophil activation, phenotypes and immunometabolism, may provide insight which leads to novel therapeutic mechanistic targets on neutrophils in IBD. It is hoped that new technologies such as single-cell RNA sequencing and spatial transcriptomics may help model pre-clinical IBD studies, improving the translation of research from murine models into clinical benefits in patients with IBD.^169^ Targeting neutrophils in human clinical trials remains complex, with attempts to reduce excessive neutrophil activation leading to a reduction in their protective physiological roles against pathogens and inflammation. Therefore, more research is needed into the complex dual role of neutrophils in intestinal health, in order to elucidate their underlying immunological involvement in the disease to realize novel therapeutic potential in IBD.
